# 3D Spheroid Cultivation Alters the Extent and Progression of Osteogenic Differentiation of Mesenchymal Stem/Stromal Cells Compared to 2D Cultivation

**DOI:** 10.3390/biomedicines11041049

**Published:** 2023-03-29

**Authors:** Anne Wolff, Marcus Frank, Susanne Staehlke, Armin Springer, Olga Hahn, Juliane Meyer, Kirsten Peters

**Affiliations:** 1 Department of Cell Biology, Rostock University Medical Centre, 18057 Rostock, Germany; 2 Medical Biology and Electron Microscopy Centre, Rostock University Medical Centre, 18057 Rostock, Germany; 3 Department Life, Light & Matter, University of Rostock, 18051 Rostock, Germany

**Keywords:** mesenchymal stem/stromal cells, 2D culture, 3D culture, spheroids, osteogenic differentiation, EDX analysis

## Abstract

Mesenchymal stem/stromal cells (MSC) are capable of progenitor cell fraction renewal or tissue-specific differentiation. These properties are maintained during in vitro cultivation, making them an interesting model system for testing biological and pharmacological compounds. Cell cultivation in 2D is commonly used to study cellular responses, but the 2D environment does not reflect the structural situation of most cell types. Therefore, 3D culture systems have been developed to provide a more accurate physiological environment in terms of cell–cell interactions. Since knowledge about the effects of 3D culture on specific differentiation processes is limited, we studied the effects on osteogenic differentiation and the release of factors affecting bone metabolism for up to 35 days and compared them with the effects in 2D culture. We demonstrated that the selected 3D model allowed the rapid and reliable formation of spheroids that were stable over several weeks and both accelerated and enhanced osteogenic differentiation compared with the 2D culture. Thus, our experiments provide new insights into the effects of cell arrangement of MSC in 2D and 3D. However, due to the different culture dimensions, various detection methods had to be chosen, which in principle limits the explanatory power of the comparison between 2D and 3D cultures.

## 1. Introduction

Mesenchymal stem/stromal cells (MSC) can renew the progenitor cell fraction or differentiate in a tissue-specific manner. In this way, MSC can adapt to specific tissue requirements. For example, MSC can differentiate into osteoblasts in bone or adipocytes in adipose tissue. In doing so, MSC contribute to tissue homeostasis and regeneration [1,2]. MSC have been identified and isolated from almost all tissues, and depending on their origin, they differ in terms of gene expression, phenotype, proliferation rate, and differentiation capacity [3]. Adipose tissue-derived MSC are referred to as adMSC and are the subject of intense research due to their great significance in regenerative therapies [4,5,6,7,8].

Osteogenic differentiation during bone formation is a complex biological process that occurs during individual development, physiological tissue turnover, or in response to a bone trauma to restore the injured area [9,10,11,12]. Bone formation requires a coordinated interaction between different cell types (among them MSC), secreted biological factors (e.g., bone morphogenetic proteins, sclerostin, and osteoprotegerin), and extracellular matrix(-bound) molecules (e.g., calcium phosphate-based inorganics in collagen matrices) to provide a supporting scaffold [10,13]. Bone formation progresses over several weeks and months [14]. MSC of various tissue origins can differentiate into progenitor cells of the osteogenic lineage and serve as a major source of osteoblasts. MSC respond flexibly to regenerative signals emanating from the surrounding microenvironment, contributing to bone homeostasis and remodeling [15]. MSC retain many of their stem cell capacities in vitro and are thus a valuable tool for molecular studies of biological factors as well as for testing pharmaceutical compounds.

Generally, two-dimensional (2D) cell cultures are used, but the 2D environment does not reflect most cell types’ rather complex structural situation in tissues (e.g., bone tissue). In 2D cultivation, aspects such as the organization of intercellular contacts or cell polarity are not properly taken into account. Therefore, three-dimensional (3D) culture systems have been developed to create more accurate physiological environments [16,17,18]. The 3D cell cultivation can be initiated in very different ways: cells can be cultivated on scaffolds for 3D arrangement, 3D spheroids can also be generated scaffold-free by placing them in hanging drops or cell clusters [18,19], or, as in this work, using a recently developed method, the magnetized cells can be assembled into defined 3D spheroids in a magnetic field, which leads subsequently to stable spheroids when further cultured in cell-repellent plates [20,21,22].

Only a few studies compare the effects of 2D vs. 3D spheroid cultivation on MSC’s osteogenic degree of differentiation. Within these few studies, the investigations are based on completely different modes of spheroid formation or MSC sources. For example, Juhásová et al., 2011, described the osteogenic differentiation of porcine adMSC within a 3D scaffold (plasma clot or plasma–alginate clot) over three weeks. Their results indicated similar osteogenic differentiation ability of porcine MSC in 2D and 3D environments, but the expression of osteogenic markers in 3D scaffolds and 2D culture grown on extracellular matrix started earlier than in the monolayers without an extracellular matrix [23]. Kabiri et al., 2012, working with human bone marrow MSC in a microwell-based 3D model, described a higher calcium and collagen type I content in 3D compared to 2D cell culture after 14 days of cultivation [24]. Son et al., 2021, described, in a microwell chip-based 3D culture model, that dental pulp-derived MSC formed 3D spheroids within 24 h, which remained stable for up to 72 h [25]. Immediately after spheroid formation, the amount of pluripotent and osteogenic markers was elevated on a gene expression level compared to control cells cultured in 2D. After 24 h, however, reduced proliferative capacity, cell cycle arrest, and increased apoptosis rates were observed in the 3D cultures [25].

Comparative analyses of osteogenic differentiation of MSC in 2D and 3D are rare, and outcomes appear heterogeneous depending on the type of MSC and the 3D model chosen. As physiological bone formation extends over many weeks, we chose a 3D spheroid model that allows precise adjustment of the number of cells in the initial spheroids and long-term culture and compared the effects of 2D and 3D cultivation of adMSC for up to 35 days. Our experiments provide new insights into the impact of cell arrangements of adMSC in 2D and 3D, respectively, as well as on differentiation status by analyzing the incorporated bone-specific calcification and the release of bone metabolism-related factors.

Studying cellular reactions such as proliferation or differentiation in 3D culture is technically demanding due to its difficult accessibility. This manuscript discusses both the challenges of 3D culturing and the limitations of direct comparison between 2D and 3D cultures.

## 2. Materials and Methods

### 2.1. Cell Culture

#### 2.1.1. Tissue Donors

The use of donor tissue received a positive ethical vote from the ethics committee of the University Medical Center Rostock. The vote is registered under the numbers A2013-0112 and A2019-0107. Cell cultures from the adipose tissue of 13 donors were used for the present study. The donor’s ages varied from 32 to 63 years, with a mean age of 46 and a gender distribution of 7.7% males and 92.3% females.

#### 2.1.2. adMSC Isolation and Cultivation

Human adipose tissue was obtained by liposuction. The harvested tissue was transported at room temperature (RT) directly or overnight to the processing site and processed after 3 to 24 h. The processing and isolation of adMSC followed the protocol of Meyer et al. [26], which was previously established by our research group. Cultivation was performed in Dulbecco’s Modified Eagle’s Medium (DMEM, Thermo Fisher Scientific, Schwerte, Germany) containing 10% fetal calf serum (FCS, PAN Biotech, Aidenbach, Germany) and 1% penicillin/streptomycin (penicillin: 100 U/mL; streptomycin: 100 mg/mL, Thermo Fisher Scientific, Schwerte, Germany, hereinafter referred to as complete culture medium). Culture medium was changed every 2–3 days.

#### 2.1.3. Two-Dimensional Cell Cultivation

For the comparative experiments, the 2D cultures were performed with a seeding amount of 6800 cells per well of a 96-well plate (≙20,000 cells/cm^2^, culture plate from Greiner Bio-One, Frickenhausen, Germany) and cultivated for up to 35 days, changing the medium every 2–3 days.

#### 2.1.4. Three-Dimensional Cell Cultivation

The 3D cell constructs (from now on referred to as 3D spheroids) of adMSC were generated using magnetizable nanobeads (n3D, Bioscience, Greiner Bio-One GmbH, Frickenhausen, Germany; nanobead diameter 50 nm, composed of gold, poly-L-lysine, and iron oxide). For this purpose, adMSC were grown to confluency at 80–90% in a 75 cm^2^ cell culture flask and incubated overnight with the magnetizable nanobeads in complete culture medium (80 µL nanobeads/12 mL complete culture medium). This incubation caused the nanobeads to attach electrostatically to the cell membrane (verified by phase contrast microscopy). The nanobead-equipped cells in the tissue culture polystyrene flask were washed twice with phosphate-buffered saline (PBS, PAN Biotech, Aidenbach, Germany) and then incubated with trypsin containing ethylenediaminetetraacetic acid/EDTA (Gibco/Thermo Fisher Scientific, Schwerte, Germany) for 5 min at 37 °C. The detached cells were suspended in a complete culture medium and centrifuged at 400 g for 5 min. After the supernatant was discarded, the cell pellet was resuspended in 5 mL of complete culture medium. The resulting cell suspensions were stored on ice until measurement. Cell suspensions were analyzed for the number of viable and dead cells with volume-calibrated cassettes (Via1-Cassette^TM^, Chemometec, Allerod, Denmark) and the NucleoCounter^®^ NC- 3000^TM^ (Chemometec, Allerod, Denmark).

The 3D spheroids were developed by seeding 100,000 nanobead-loaded cells per well in cell-repellent plates (Cellstar 96-well plate, Greiner Bio-One, Frickenhausen, Germany). Immediately after that, the plates were exposed to a magnetic field that was installed below the plate. This way, the 3D spheroids with a defined cell number were formed overnight. After spheroid formation, the magnetic field was removed, and the 3D spheroids were cultured for up to 35 days. To avoid the loss of spheroids during the medium change every 2–3 days, the spheroids were briefly re-exposed to the magnetic field during the medium changes.

#### 2.1.5. Osteogenic Stimulation

Osteogenic stimulation was performed three days after the respective 2D and 3D cultures were initialized. For this purpose, the following substances were added to the complete culture medium (see Section 2.1.2): 0.25 g/L ascorbic acid, 1 μM dexamethasone, and 10 µM β-glycerophosphate (osteogenic stimulating compounds from Sigma-Aldrich Chemie GmbH, Taufkirchen, Germany). The unstimulated cultures (i.e., complete culture medium without the mentioned osteogenesis-stimulating additives) were prepared in parallel.

### 2.2. Phenotyping of 3D Culture Spheroids

Spheroids were visualized with phase contrast microscopy in an appropriate culture medium (*n* = 4, Zeiss Axiovert 25, 10× objective; Carl Zeiss Microscopy Deutschland, Oberkochen, Germany) after initial spheroid formation and after 14 and 28 days under unstimulated and osteogenic cultivation conditions. To determine the diameter of the spheroids, the phase contrast images (Zeiss Zen Blue, Carl Zeiss Microscopy Deutschland, Oberkochen, Germany) were converted to binary data using ImageJ [27], and the diameter of each spheroid was calculated.

### 2.3. Determination of Cell Numbers and Cell Diameter

The NucleoCounter^®^ NC-3000™ (Chemometec, Allerod, Denmark) assay for aggregated cells was used to determine cell numbers of adMSC after different cultivation approaches. Cells were incubated with trypsin/EDTA at 37 °C (2D culture: 5 min; 3D culture: 20 min), dissociated, and the detachment/disintegration reaction was stopped with the same volume of culture medium containing FCS (*n* = 4). The obtained cell suspensions were stored on ice until measurement. Cell number and cell diameter were measured, and the number of viable and dead cells were analyzed using volume-calibrated cassettes (Via1-Cassette^TM^) and the NucleoCounter^®^ NC-3000^TM^ (both Chemometec, Allerod, Denmark) according to the manufacturer’s instructions.

### 2.4. Vital Staining and Image Analysis

After 35 days under corresponding stimulation, the 2D and 3D cultures were stained with calcein acetoxymethyl ester (calcein AM, AAT Bioquest, Sunnyvale, CA, USA), propidium iodide (PI, Thermo Fisher Scientific, Schwerte, Germany), and 2′-(4-Ethoxyphenyl)-6-(4-methylpiperazin-1-yl)-1H,3′H-2,5′-bi-1,3-benzimidazole (Hoechst 33342, Sigma-Aldrich GmbH, Taufkirchen, Germany) to determine their viability. For this purpose, cells were washed with PBS and then incubated in a complete culture medium containing calcein AM 1:3000, PI 1:50, and Hoechst 33342 1:2000 for 20 min at 37 °C. After incubation, samples were washed and visualized in PBS on the inverted confocal laser scanning microscope LSM 780 (Carl Zeiss Microscopy Deutschland, Oberkochen, Germany). Images were taken and analyzed using ZEN 2011 software (black edition, Carl Zeiss Microscopy Deutschland, Oberkochen, Germany). For the 3D spheroids, several images were acquired with the Z-stack function (slices with an interval of 5 µm, pinhole 1 AU), and a 3D reconstruction was created by an overlay. The overlay was used to illustrate the distribution over the entire spheroid (*n* = 3).

### 2.5. Determination of Osteogenic Differentiation in 2D Cultures

To assess the mineralization of the adMSC, the fluorescence-based in vitro mineralization OsteoImage^TM^ assay (Lonza Biosciences, Basel, Switzerland) was used, which binds to the hydroxyapatite portion of the deposited bone-like nodules in the cells. According to the manufacturer’s instructions, cells were washed with PBS without Mg^2+^ and Ca^2+^ and then incubated with 4% PFA for 10 min at RT. This was followed by a washing step. The staining solution was subsequently added at a dilution of 1:100 for 30 min at RT in the dark. After the incubation step, the cells were washed four times, leaving the wash solution on the cells at the end. Images were acquired using Observer Z1 (Zeiss Microscopy Deutschland, Oberkochen, Germany) and ZEN 2011 software (black edition, Zeiss Microscopy Deutschland, Oberkochen, Germany).

### 2.6. Determination of Alkaline Phosphatase (ALP) Activity

The ALP activity was visualized by exposure to a solution of 67 mM 2-Amino-2-methyl-1.3-propanediol (AMPED), 2.7 mM Naphthol AS-MX phosphate, and 2.7 mM Fast Red Violet LB Salt in H_2_O. For this purpose, cell cultures were washed twice with PBS without Mg^2+^ and Ca^2+^, fixed with 4% PFA for 5 min, followed by a washing step with PBS without Mg^2+^ and Ca^2+^. The previously prepared staining solution was added and incubated for 10 min. Subsequently, the cell cultures were washed once with PBS without Mg^2+^ and Ca^2+^ and imaged using a microscope (Zeiss Zen Blue).

### 2.7. Preparation of Microscopic Slides for Histological Analysis

The 3D spheroids were washed with PBS after 14 and 28 days of cultivation and fixed in 4% paraformaldehyde (PFA) at 4 °C overnight (*n* = 4). After washing again, samples were dehydrated in 30%, 50%, 70%, and absolute ethanol for 30–60 min each. The samples were stored in absolute ethanol until embedding in LR White acrylate resin (medium grade, Plano GmbH, Wetzlar, Germany). For embedding, the specimens were infiltrated with a 1/1 mixture of ethanol/LR White in an open vial after dehydration overnight. Subsequently, infiltration with pure LR White was performed for four hours. Samples were transferred to gelatin capsules, filled with LR White, hermetically sealed, and polymerized at 50 °C for approximately two days. After embedding in LR White resin, the spheroids were exposed from the blocks with the help of a trimming mill (Leica EM Trim 2, Leica Microsystems GmbH, Wetzlar, Germany), and thin sections with a section thickness of 0.5 µm were prepared with an ultramicrotome (Ultracut S, Reichert/Leica, Vienna, Austria) using a diamond knife (Diatome, Nidau, Switzerland). Care was taken to yield cross-sections close to the maximum diameter of the spheroids for analysis. These sections were stained with toluidine blue and covered in mounting medium for analysis with a light microscope (Zeiss Axioskop 40) equipped with a digital camera (Zeiss Axiocam ERc 5s, Zeiss Microscopy, Oberkochen, Germany).

### 2.8. Energy Dispersive X-ray Spectroscopy (EDX): Analysis of Calcification

Following semi-thin sectioning with the ultramicrotome described above, the polished resin blocks with the remaining halves of the respective spheroids were processed further for analytical scanning electron microscopy using energy dispersive X-ray spectroscopy (SEM-EDX) in a ‘block-face mode’. For this analysis, the resin blocks were mounted on a heavy metal-free Al-SEM carrier (Plano Wetzlar, Wetzlar, Germany) with adhesive conductive carbon tape (Spectro Tabs, Ted Pella Inc., Redding, CA, USA) and were coated with an ultrathin carbon layer (5.0 nm) under high vacuum conditions to establish sample surface conductivity (CCU 010 HV-Coating Unit, Co. Safematic GmbH, Zizers, Switzerland). Samples were analyzed by a field emission scanning electron microscope (FE-SEM, Merlin VP Compact, Zeiss, Oberkochen, Germany) equipped with an EDX-detector (XFlash 6/30, energy resolution 126 eV, Bruker, Berlin, Germany) for the detection of element-specific X-rays. Representative areas of the samples were analyzed by secondary electron (SE)-imaging, collecting topographic and material contrast information from the specimens. In addition, information on the elemental composition and distribution over defined sample areas was collected with the EDX-detector operated in the spectrum and element mapping mode using Bruker Quantax Esprit Microanalysis software (version 2.0). The presence of calcium, phosphorus, and other elements was analyzed according to their characteristic X-ray emissions and compared specifically after 14 and 28 days of differentiation in both conditions (*n* = 3). Further details on the respective analysis conditions applied are specified in the figure legends.

### 2.9. Quantification of Bone Metabolism-Affecting Factors by Multiplex Analysis

Analyses were performed on several bone metabolism-affecting factors. Dickkopf 1 (DKK1), osteoprotegerin (OPG), interleukin 6 (IL-6), leptin, sclerostin (SOST), osteocalcin (OC), osteopontin (OPN), insulin, fibroblast growth factor (FGF23), adrenocorticotropin (ACTH), interleukin 1β (IL1β), parathyroid hormone (PTH), and tumor necrosis factor (TNFα) were quantified in both supernatants and lysates of 2D- and 3D-cultured adMSC using the MILLIPLEX^®^ Human Bone Magnetic Bead Panel—Bone Metabolism Multiplex Assay (EMD Millipore Corporation, Billerica, MA, USA) according to the manufacturer’s instructions. The samples were collected after 7 and 28 days from unstimulated and osteogenically stimulated cultures, respectively. Lysates were prepared using the Bio-Plex^®^ Cell Lysis Kit (Bio-Rad, Feldkirchen, Germany). Briefly: standards, controls, and samples were incubated with the antibody bead mix in a 96-well plate at 4 °C for 16–18 h. The plate was then incubated for 1 h with detection antibody at RT, followed by 30 min incubation with streptavidin-phycoerythrin at RT. Fluorescence intensity was measured using the Bio-Plex^®^ 200 System (Bio-Rad, Feldkirchen, Germany) and Bio-Plex^TM^ Manager 4.1.1 software (Bio-Rad). Protein concentrations were normalized to the respective cell number measured with NC-200^TM^ (*n* = 4).

### 2.10. Statistical Analysis

Sample size *n* included 4 donors in all statistical analyses. Depending on the assay and the associated accuracy, each sample was measured in duplicate or triplicate technical replicates. Microsoft Excel 2016 and GraphPad Prism 9 software were used for statistical analysis. Data were presented as median values in the form of boxplots. A two-way ANOVA post hoc uncorrected Fisher’s LSD was used to analyze spheroid diameter, cell number, cell diameter, and protein amount. The probability value of *p* < 0.05 was set as a significant difference (indicated by ^§^, ^#^, and * in the graphs). All graphs were created with GraphPad Prism 9 software.

## 3. Results

### 3.1. Spheroid Morphology

**Figure 1:**
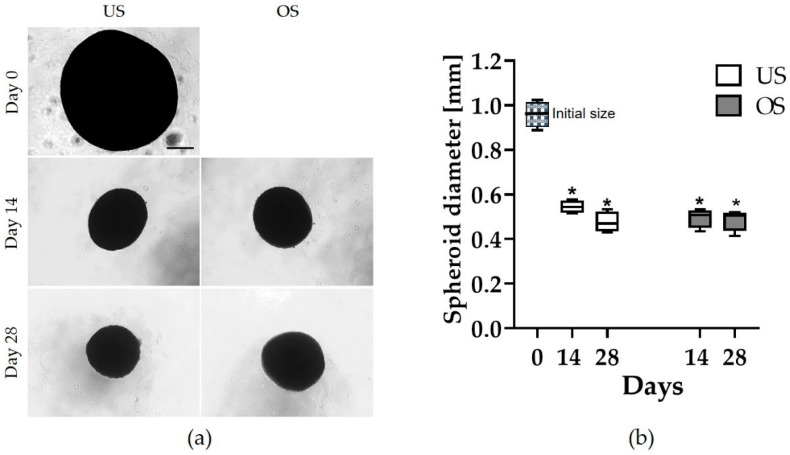
Spheroid morphology and analysis of the spheroid diameter. (**a**) Unstimulated (US) and osteogenically stimulated (OS) spheroids initially after spheroid formation (day 0) and after 14 and 28 days (*n* = 4, phase contrast, Axiovert 25, scale bar: 200 μm); (**b**) Analysis of the spheroid diameter of unstimulated and osteogenically stimulated spheroids at day 0 and after 14 and 28 days of cultivation (* significantly different from day 0, *p* < 0.0001; one-way ANOVA post hoc uncorrected Fisher’s LSD, with *n* = 4 for diameter measurement by image analysis using ImageJ).

The 3D spheroids, each generated from 100,000 adMSC, were formed after a few hours by exposure to a magnetic field and were examined for up to 35 days. Phase contrast microscopy was used to analyze the spheroid diameter at different time points (Figure 1a). Within the first day after the initiation of the experiment, a uniform round spheroid with a diameter of about 0.96 mm was observed (day 0). Within the next few days of cultivation, there was a significant reduction in the size of the spheroids, which decreased to 0.56 mm after 7 days in the unstimulated spheroids and to 0.53 mm in the osteogenically stimulated spheroids (see Supplemental Figure S1). In the following weeks, the size of the unstimulated spheroids decreased only slightly (0.54 after 14 days and 0.47 after 28 days) or remained constant for the osteogenically stimulated spheroids (Figure 1b).

### 3.2. Cell Numbers and Cell Diameters in 2D and 3D Culture

**Figure 2:**
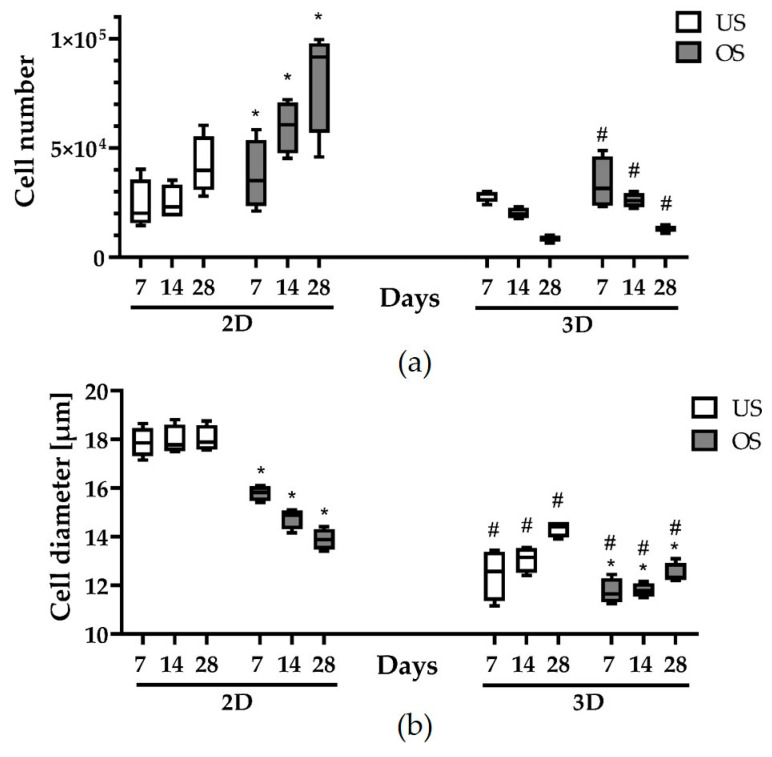
Quantification of the cell number and cell diameter of adMSC in 2D and 3D cultures up to 28 days. (**a**) Cell number of unstimulated (US) and osteogenically stimulated (OS) adMSC over 28 days (*n* = 4; * significantly different from the US at the respective time point and dimension (2D/3D), * *p* < 0.0001; ^#^ significantly different from 2D at the respective time point and stimulation (US/OS), two-way ANOVA post hoc uncorrected Fisher’s LSD; ^#^ *p* < 0.0001). (**b**) Diameter of single cells under US and OS conditions in 2D and 3D culture (*n* = 4; * significantly different from the US at the respective time point and dimension (2D/3D), * *p* < 0.05; ^#^ significantly different from 2D at the respective time point and stimulation (US/OS), two-way ANOVA post hoc uncorrected Fisher’s LSD; ^#^ *p* < 0.0001).

To gain an overview of the number of cells in the course of cultivation, these were counted over the experimental period, and the cell diameter was analyzed in each case (Figure 2a). There was a clear difference between the number of cells in the 2D culture and those in the 3D spheroid culture. While the cell quantity in the 2D culture showed a clear increase within the first 7 days (from the initial 6800 cells/well to approx. 20,000/well), the cell quantity in the 3D spheroids decreased significantly (from initially 100,000 cells/spheroid to approx. 20,000 cells/spheroid after 7 days). Over the analysis period of 28 days, the cell number in the 2D culture increased markedly (especially with osteogenic stimulation), whereas the cell number in the 3D spheroids continued to decrease. In the 3D spheroids, there was no significant difference in cell number between the unstimulated and the osteogenically stimulated spheroids (Figure 2a).

The analysis of the individual cell diameter led to a different situation when comparing unstimulated and osteogenically stimulated 2D and 3D spheroid cultures (Figure 2b). While the cell diameters in the unstimulated 2D cultures remained constant over the analysis period of 28 days (at approx. 18 µm), in 2D culture there was a significant reduction in cell diameter to around 16 µm after just 7 days of cultivation under osteogenic stimulation, which decreased over the next 3 weeks to a diameter of about 14 µm. Cells in the 3D spheroid cultures were significantly smaller (size range between approx. 12 and 15 µm). Interestingly, after the early size reduction after 7 days, the cell size increased again slightly over the next 3 weeks. Unlike in the 2D culture, the osteogenic stimulation did not lead to a significant cell size change in 3D spheroids (Figure 2b).

### 3.3. Live/Dead Staining of Unstimulated and Osteogenic-Stimulated Spheroids and 2D Cultured adMSC

**Figure 3:**
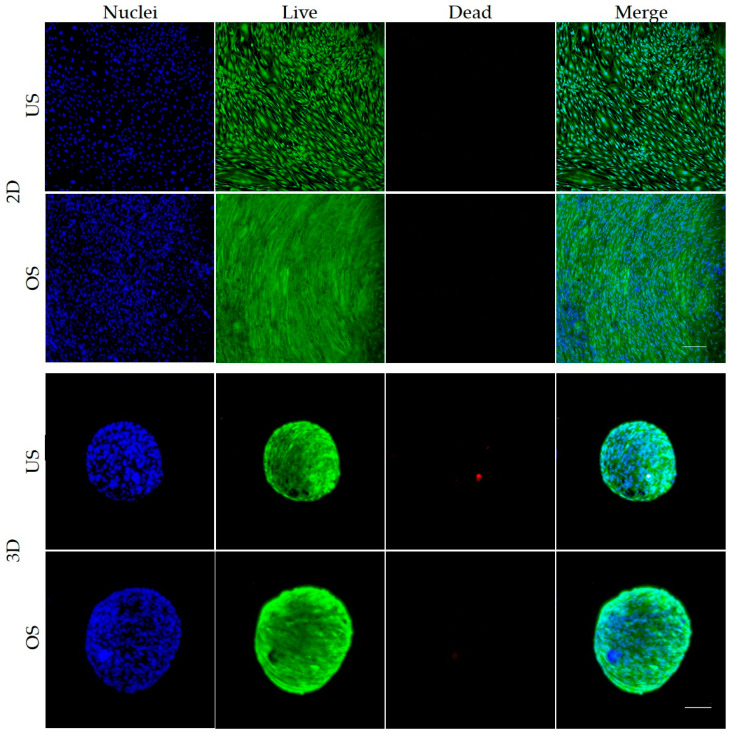
Live/dead staining of unstimulated (US) or osteogenically stimulated (OS) adMSC in 2D culture (**top** panels) and 3D spheroid culture after 35 days (**bottom** panels) (exemplary images of *n* = 4, LSM 780, Zen black software, overlay of Z-stacks, green: live cells, red: dead cells, blue: nuclei; scale bar: 200 µm).

In addition to the indications of cell viability and cell death, the live/dead staining also allows a clear representation of the cell arrangement (Figure 3). Consistent with the previous determination of cell number, the vital staining also showed a higher cell number after osteogenic stimulation compared to the unstimulated cells after 35 days in the 2D culture (Figure 3). In the 2D culture, the shape of the osteogenically stimulated cells was narrower and more spindle-shaped than that of the unstimulated cells. Little to no dead cells were detected in the 2D culture under either condition.

The 3D spheroids also showed mainly live cells (green) and evenly distributed nuclei (blue) over the entire spheroid after 35 days of cultivation, both without and with osteogenic stimulation. A few dead cells (red) could be detected in both conditions. These were not locally confined to one area. As the diameter determination already showed, a smaller diameter of the unstimulated spheroid compared to the osteogenic spheroid could also be seen here. In the 3D model, the cells were arranged in a whirl-like manner. Since only the outer shell could be analyzed with this method, further investigations of the inner layers and the spheroid core followed using ultrathin sections.

### 3.4. Analysis of Organization and Structure of adMSC in 3D Spheroid Culture by Histological Sections

To investigate the cellular organization and structure of adMSC arranged in 3D in greater detail, we further examined semi-thin sections of the spheroids embedded in resin. Sections with a thickness of 0.5 µm were stained with toluidine blue, which provided excellent resolution in the Z-axis and an overview of the structures.

**Figure 4:**
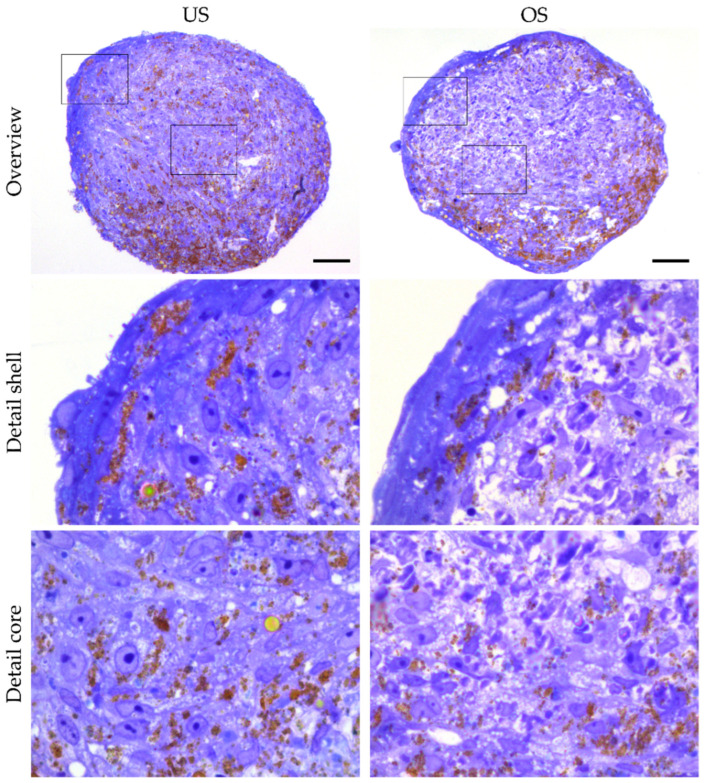
Representation of 3D spheroids by overview staining of thin sections with toluidine blue. Overview images of an unstimulated (US) spheroid and an osteogenically (OS) stimulated spheroid after 14 days (**top** panel). Frames in the overview images correspond to the areas shown below in high magnification. Details of the shell and core regions of the same spheroids show differences in cell arrangement, cell shape, and cellular spacing (**middle** and **bottom** panels) (scale bars represent 50 µm for the overviews and 10 µm for the detailed magnifications, respectively).

Microscopic analysis of the sectioned spheroids showed a regular outer lining of the spheroids arranged in a thin shell region with densely packed cells around an elaborated core region after 14 days of cultivation (Figure 4). Higher magnification with oil immersion showed that both the unstimulated and the osteogenically stimulated spheroids formed these two zones with different cell arrangements (Figure 4). In both treatments, the outer, enveloping layer was characterized by cells with a flat, more elongated phenotype in a compact arrangement (Figure 4). In contrast, the cells localized inside the spheroids exhibited a rounder shape with relatively large nuclei and prominent nucleoli. They basically showed a dense arrangement, with the spacing of the cells in the core region being greater than in the shell region. In the core region of the spheroids, another difference was revealed with respect to the compactness of the cellular arrangement: while the compactness of the unstimulated spheroids was relatively high, the osteogenically stimulated spheroids appeared more loosely packed after 14 days. Numerous cell-free matrix areas were observed to be evenly distributed, providing space for additional extracellular deposition and calcification (Figure 4). This organization of the spheroid was maintained almost over the entire observation period of 35 days. A necrotic spheroid core was never recognizable. Under both stimulation conditions, accumulations of the nanobeads used to develop the spheroids were visible in some areas (brown coloring) and tended to form larger clusters, especially near the shell region. After a cultivation period of 28 days, the spheroids appeared more fragile, and there was an increased accumulation of nanobeads with a concomitant impression of a decrease in cell density, especially in the osteogenically differentiated spheroids. The decrease in cell density could be related to an increase in calcification, which makes these spheroids appear more compact (see Supplementary Figures S2 and S3).

### 3.5. Osteogenic Differentiation in 2D Culture

**Figure 5:**
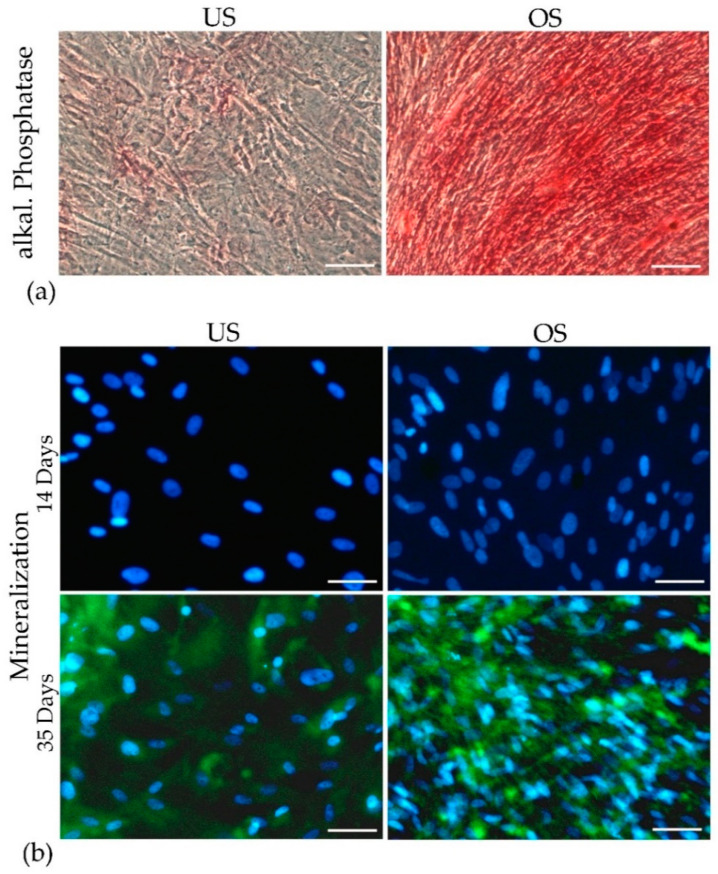
Osteogenic differentiation of adMSC in 2D culture. (**a**) Alkaline phosphatase staining in unstimulated cultures (US) and after osteogenic stimulation (OS) after 21 days. (**b**) Staining of mineralization by OsteoImage^TM^ after 14 and 35 days in US cultures and in OS cultures (Observer Z1, Zeiss, scale bar: 100 µm).

The potential for osteogenic differentiation of adMSC in 2D culture was analyzed by detecting alkaline phosphatase activity after 21 days of cultivation without stimulation (US) and with osteogenic stimulation (OS) (Figure 5a). After 21 days, slight red staining was observed in the unstimulated cell culture, which was limited to a few cells (mainly in the more clustered areas). In contrast, osteogenic stimulation resulted in distinct red staining encompassing the entire culture.

The detection of mineralization was negative after 14 days of cultivation (for both US and OS cultures), while it was positive after 35 days of cultivation for both culture conditions (Figure 5b). However, the staining intensity was markedly higher in the case of osteogenic stimulation. At the same time, the number of cells (recognizable by the number of cell nuclei visible) was also clearly higher under osteogenic stimulation.

### 3.6. Osteogenic Differentiation in 3D Spheroid Culture

The detection of osteogenic differentiation in 3D spheroid culture could not be performed in the same way as the analysis in 2D due to the embedding in a resin and the associated denaturation. For the evaluation of the 3D culture, the resin-embedded spheroids were used after cutting the histological sections and were subjected to SEM-EDX analysis on the surface of the resin blocks. The combined use of these techniques in the so-called ‘block-face’ approach allows both an overview of the structures present in the samples and precise mapping of their elemental composition. Due to inherent interactions with the incident electron beam, element-specific X-rays are produced from the specimen, e.g., from osteogenic matrix depositions containing calcium and phosphorus.

**Figure 6:**
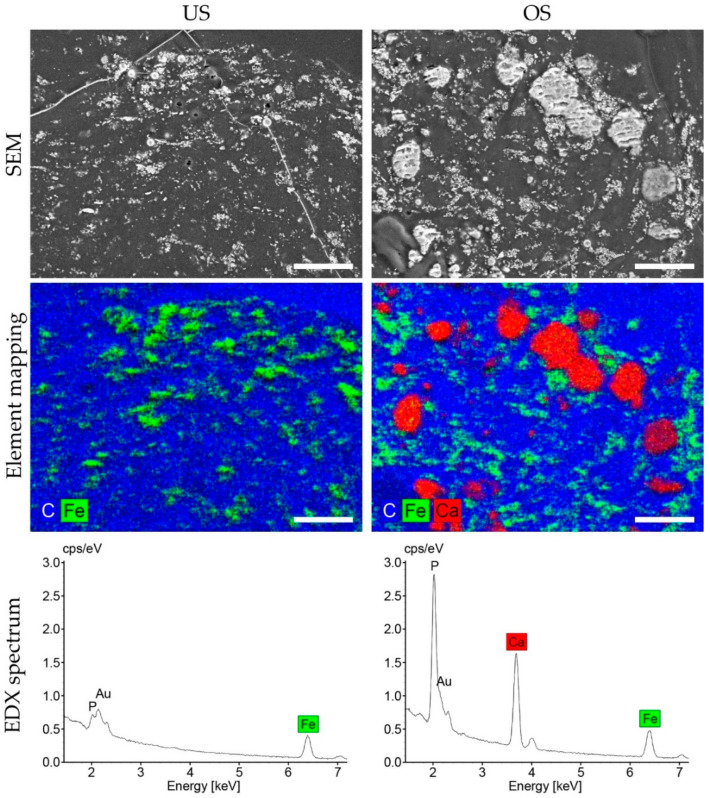
SEM-EDX analyses of unstimulated and osteogenically stimulated adMSC in 3D spheroid culture after 14 days. The surface planes exposed after cutting for histological analysis from the embedded unstimulated spheroids (US) or osteogenic-stimulated spheroids (OS) were investigated with a secondary electron detector. This reveals metal particles and deposited calcified matrix as brighter areas within the spheroids (**top** panel). Combined mapping with X-ray spectroscopy shows the specific element distribution across the samples. Iron particles (green) and calcium-containing deposits (red) are located in a carbon-rich matrix of cells and resin (blue), as outlined by the corresponding color coding (**middle** panel). Spectra recorded from the area during mapping (acquisition time: 6 min) show characteristic element peaks for calcium and phosphorus only in the osteogenically stimulated spheroids, in line with the observation that unstimulated spheroids lack calcium phosphate matrix deposition (**bottom** panel). Similar amounts of iron, i.e., traces of the magnetic nanobeads used for spheroid generation, are found in the spectra of both samples (scale bars: 30 µm).

With the help of this method, mineralized structures could be highlighted on the respective sample surface in combination with SEM imaging (Figure 6, top and middle panels). In addition, the element spectrum recorded simultaneously allows for a direct semi-quantitative comparison between differentially stimulated spheroids (Figure 6, bottom panels). Mineralization (more precisely, calcification) could already be detected after 14 days of osteogenic stimulation of the 3D spheroids, which did not occur in the unstimulated state. Large electron-dense plaques were visible in the SEM images of the osteogenic stimulated spheroids, and EDX analysis clearly showed that these structures were calcium-rich in contrast to the finely dispersed granular structures. The latter was also present in the unstimulated 3D spheroids, where the EDX spectrum showed only the presence of iron and some small amounts of gold. These metals are traces from the magnetic nanobeads used for spheroid generation and were also detectable in the osteogenically stimulated 3D spheroids with a similar distribution. After 28 days of cultivation, a relatively similar result was obtained (see Supplemental Figure S2). It should be noted that the calcium phosphate enrichment of the osteogenically stimulated spheroids could not only be demonstrated in selected areas by SEM-EDX mapping but, using EDX spectrum measurements, was detectable in analyses of the entirety of the cross-section surfaces (see Supplemental Figure S3).

### 3.7. Release of Bone Metabolism-Affecting Factors in 2D and 3D Spheroid Culture

**Figure 7:**
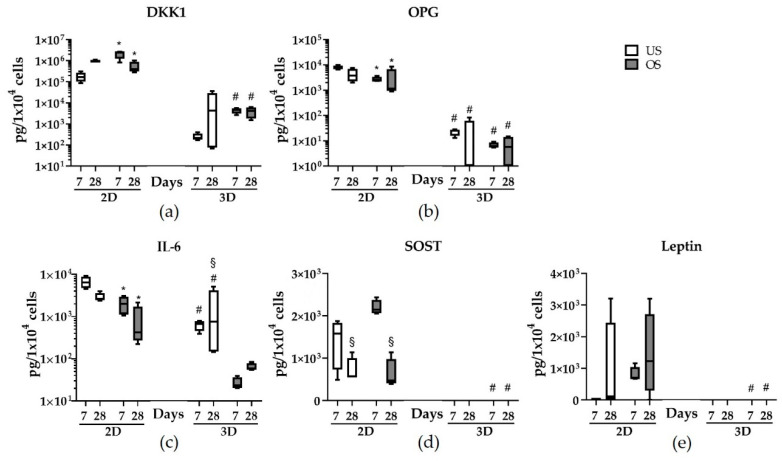
Quantification of bone metabolism-affecting factors released from unstimulated (US) and osteogenically stimulated (OS) 2D and 3D spheroid cultures after 7 and 28 days. (**a**) DKK1; (**b**) OPG; (**c**) IL-6; (**d**) leptin; (**e**) SOST (concentrations were normalized to cell number, *n* = 4; ^#^ significantly different from 2D cultures at the respective time point; * significantly different from day 7 under the same cultivation conditions; ^§^ significantly different from US culture at the respective time point and the dimension of cultivation; one-way ANOVA and two-way ANOVA, post hoc uncorrected Fisher’s LSD; ^#^ *p* < 0.05; * *p* < 0.05; ^§^ *p* < 0.05).

The amount of bone metabolism-affecting factors was analyzed in the supernatants and the lysates of the 2D and 3D adMSC cultures by multiplex analysis. The utilized multiplex assay allowed the measurement of 13 analytes in the same sample. The marker concentrations in the supernatants were consistently higher than in the lysates, so only the release of factors in the cell culture supernatant is depicted here (Figure 7 and Table 1, osteogenic marker concentrations from cell lysates in Supplemental Table S1).

Of the thirteen analytes investigated, nine could be detected in the different adMSC cultivation settings, namely DKK1, OPG, IL-6, SOST, leptin, OPN, OC, insulin, and FGF23 (four factors were not found: PTH, IL1β, ACTH, and TNFα). The release pattern showed dependencies on the culture dimension and the stimulation mode; the release of the factors DKK1, OPG, and IL-6 was generally higher in the 2D culture than in the 3D spheroid culture (Figure 7 and Table 1). SOST, leptin, OPN, OC, insulin, and FGF23 were released from the 2D-cultured adMSC but not from the 3D spheroids. In the case of DKK1, for example, there was increased release due to osteogenic stimulation (Figure 7a and Table 1). This effect was also observed in the 3D culture but was not as pronounced.

OPG release was significantly lower in the 3D spheroid culture and was reduced by osteogenic stimulation in both the 2D and 3D spheroid cultures (Figure 7b and Table 1). The release of the pro-inflammatory factor IL-6 was decreased in the 3D culture and was substantially reduced by osteogenic stimulation in both the 2D and 3D spheroid cultures (Figure 7c and Table 1). SOST and leptin were only detectable in the 2D culture supernatants; while SOST release was reduced over time, there was an increase in leptin release, especially in osteogenically stimulated 2D cultures.

## 4. Discussion

Cell culture models are a fundamental tool for biomedical research that can reduce the number of animal experiments [28,29,30]. To date, most of these tests have been performed in 2D cultures. However, 2D cell culture models do not represent the complex microenvironment, and consequently, the induced cell response shown in 2D culture does not correspond to the physiological or biological response to the molecules [30]. Because of these limitations of culturing cells in 2D, 3D cell culture models are a growing area of research that should offer several advantages over 2D culture; 3D cultures are structured differently regarding cell–cell and cell–extracellular matrix interactions, resulting in altered inter- and intracellular communication. In addition, the 3D arrangement changes the accessibility of nutrients and oxygen, which is at least more similar to the physiological tissue situation than can be the case in a 2D cell arrangement [31]. However, in addition to the cell culture dimensionality, the detailed cell culture conditions are crucial for the behavior of the cells in terms of proliferation, differentiation, and other specific cellular reactions, such as the release of biologically active factors, the synthesis of the extracellular matrix, and cell–cell interactions. The cultivation conditions are determined by the addition of specific soluble components (e.g., growth factors, hormones, and chemical compounds, respectively) and insoluble substrates (e.g., artificial, semi-artificial, and natural) [29,32,33]. The outcome of the complex interactions also depends on the concentration, the timing of the stimulation, and the specific combination of factors. It must therefore be assumed that all the aspects mentioned are likely to contribute to large differences in the resulting outcomes, making it even more difficult to draw precise conclusions about the examined aspects in the biological/physiological situation. Therefore, only an accurate, well-described, and comprehensive analysis can improve the transferability of the results.

As osteogenic differentiation in 3D culture is comparatively sparsely described, there is still a need for research on this topic. Therefore, in this study, we compared the effects of 2D and 3D cultivation of human adMSC without specific stimulation and with osteogenic stimulation, respectively, focusing on differentiation and the release of bone metabolism-affecting factors. In the 3D model system used in this study, the spheroid formation was initiated by equipping the adMSC with cell-binding magnetizable nanobeads and then placing them in a magnetic field overnight. After completion of the spheroid formation, the spheroid is stable in a low-binding cell culture plate without magnetic field exposure. The application of this technique resulted in the formation of spheroids with a uniformly defined size from the beginning of the experiment. Applying the magnetic field during the media exchange also prevents the loss of the spheroid due to aspiration. Various studies have shown that the nanobeads used are biocompatible and have no effect on cell development in terms of viability, proliferation, or metabolism [22,34,35]. As described in these reports, we also did not observe any negative effects during cultivation (not even in the areas where the nanobeads accumulated), so we continued our experiments for up to 35 days. During this relatively long study period (to our knowledge, most 3D culture observations are terminated earlier, e.g., [23,25,36,37]), the cell viability was comparatively high in both the 2D culture and the 3D spheroids, and different specific changes occurred depending on stimulation and culture size.

### 4.1. Morphology of Unstimulated and Osteogenically Stimulated MSC Spheroids

The adMSC equipped with magnetizable nanobeads formed stable 3D spheroids in the magnetic field after a few hours. The spheroid size decreased significantly during the first 7 days of cultivation (from approx. 0.96 mm to approx. 0.56 mm for unstimulated spheroids and 0.53 mm for osteogenic stimulated spheroids). In the following 4 weeks of follow-up (from day 7 to day 35), there was no further significant reduction in spheroid size. The additional osteogenic stimulation did not induce significant size differences. This distinguishes them from tumor spheroids, where an increase in size during the cultivation period is typical [38].

However, after about 14 days, there was some difference in the internal spheroid structure of unstimulated and osteogenically stimulated 3D spheroids (high cell compactness in the unstimulated spheroids, more loosened appearance in the osteogenically stimulated spheroids), probably due to increased intercellular spacing associated with matrix deposition and the onset of mineralization with calcium phosphate agglomerates (discussed in Section 4.2). The 3D spheroids from adMSC did not develop a necrotic core over the entire observation period of 35 days under any of the selected cultivation situations. This is in line with several studies that have described the absence of this necrotic core in 3D-cultivated MSC precisely [22,39,40]. The reason for the absence of cell death in 3D spheroids developed from MSC is suggested to be a cell type-specific, efficient counter-regulation of oxygen deficiency via hypoxia-inducible factor 1 subunit α (HIF1α) and other factors such as increased levels of heme oxygenase 1 [39,40]. There is a clear difference in cell survival of the core cell population between spheroids from the relatively slowly proliferating MSC and spheroids from rapidly proliferating tumor cells, in which cell death or necrosis, respectively, in the spheroid core is consistently described [41]. Using adMSC (the cell type also used in our study), Schmitz et al. [42] showed that not only the spheroid size but also the spheroid cultivation technique plays a decisive role in the occurrence of critical hypoxia. They showed large differences in spheroid size depending on the spheroid cultivation method (from 0.18 mm on microstructured plates to 0.92 mm spheroids in the hanging drop technique). The stabilization of HIF-1α could be demonstrated at the earliest from a spheroid size of 0.6 mm. The spheroids presented in our work were stable below 0.6 mm after a 7-day cultivation in both cultivation methods, so there should be no critical oxygen undersupply and, thus, no trigger for cell death in the core of the spheroids.

There is, however, evidence that not only the number of cells but also the size of the cells can influence the size of the spheroid. The differences we observed in the diameters of the individual cells in the different cultivation methods were significant. The diameter of the cells in the 3D cultures was, on average, about 30% smaller than in the 2D culture. This is consistent with several studies in which a reduction in cell volume was observed with 3D cultivation [22,43,44,45]. The decrease in cell size in 3D culture is likely caused by changes in the organization of the cytoskeleton, as actinomyosin and the tension generated by myosin have been shown to play a central role in the process of 3D spheroid formation [43].

### 4.2. Osteogenic Differentiation and Release of Bone Metabolism-Affecting Factors from adMSC in 2D and 3D Culture

The detection of osteogenic differentiation can be performed at different levels. Osteogenesis-typical signal transduction, osteogenesis-associated factors, enzymes, and osteogenesis-typical extracellular matrix can be determined at transcriptional, protein, and chemical levels [46,47]. However, not all methods were applied in the same way in the two in vitro culture models. While the usual detection methods, such as antibody-mediated detection and enzymatic staining methods, could be used in 2D culture, the detection methods in 3D spheroid culture could only be applied to a limited extent, and alternative, and usually more complex, methods had to be used. Due to the compactness of the spheroids and the comparatively substantial nanoparticulate content, the spheroids were embedded in a rigid resin. The resin embedding allows the production of thin sections so that optical resolution at the level of single cells is possible. In addition, the stiffness of the resin enables thin sections to be made without distortions left by the metallic nanobeads in softer materials such as paraffin. However, the resin embedding hinders specific antibody binding and the detection of bone-specific alkaline phosphatase activity. Therefore, the comparative study of osteogenic differentiation of adMSC in 2D and 3D cultures was performed based on different methods, which limited the explanatory power. While adMSC in the 2D culture showed noticeable mineralization only after 35 days of cultivation (detected by OsteoImage^TM^), mineralization in 3D spheroids, detected by EDX analysis, took place significantly earlier, namely after 14 days, and persisted over the observation period of up to 35 days. The areas of mineralization accumulating over the investigation period give the impression of lower cell counts with the same spheroid size. This is consistent with the impression of mature bone tissue, in which the mineralization fraction is often greater than 50% [48]. Since osteogenic differentiation reached the center of the osteogenically differentiated spheroid, we assume that the differentiation compounds (i.e., dexamethasone, ascorbic acid, and β-glycerophosphate) penetrated the complete spheroids.

The number of studies comparing osteogenic differentiation of MSC in 2D and 3D cultures is relatively low, which may also be due to the aspects mentioned in the section before. It is reported in these studies that osteogenic differentiation starts earlier in 3D than in 2D culture [23,24,25]. Furthermore, there are indications that coating the culture surface with extracellular matrix molecules also increases osteogenic differentiation [23,49,50]. Thus, a physiological extracellular matrix and 3D cell contact may have a positive effect on osteogenic differentiation.

In contrast to the earlier and more abundant mineralization in the 3D spheroid culture, the release of factors affecting bone metabolism was continuously significantly reduced in the 3D culture. This was more evident for some factors than others; for example, the release of DKK1 and SOST into the cell culture supernatant, proteins that inhibit the Wnt signaling pathway, was significantly reduced or undetectable in 3D spheroids after 7 days of culture compared to 2D culture. Since the release of both factors, DKK1 and SOST, is shown to have an inhibitory effect on bone formation [51,52], the reduced release and increased mineralization in 3D spheroid culture are consistent. OPG, a cytokine receptor that influences bone density in the organism, is also reduced by more than a thousand-fold in the 3D culture compared to the 2D culture. The reduced release of IL-6 in the 3D culture is consistent with other studies [22,44,53]. It is noticeable that IL-6 release is lower during osteogenic stimulation than in the unstimulated state (both in 2D and 3D). Since, to our knowledge, this aspect has yet to be investigated in this context, we can only speculate here. For instance, the glucocorticoid dexamethasone required for osteogenic stimulation and not present in the unstimulated cultures could influence the inflammatory status of adMSC, resulting in reduced IL-6 release [54]. It is also striking that leptin, OPN, and OC could only be detected in the supernatants of the 2D cell culture but not in the supernatants of the 3D culture.

The fundamental reduction in soluble factors affecting bone metabolism was surprising, given the robust osteogenic differentiation that occurred in the 3D spheroid cultures during this 35-day experimental period. Some studies have described the increased release of growth factors and osteogenesis-related factors when MSC were cultured in 3D. However, these studies focused on time points much earlier than those chosen in our study (e.g., up to 96 h) [25,36]. Therefore, there is limited evidence of soluble factor release over a more extended time period, and we hypothesize that 3D spheroids in long-term culture can maintain the soluble factor communication required for osteogenic differentiation at a low level because communication occurs through the three-dimensionally organized cell–cell or cell–matrix contacts. However, further studies are needed to show whether, for example, specific osteogenic preconditioning of MSC prior to their placement in the 3D spheroid or alternative 3D models, e.g., scaffold-based models that mimic the natural microenvironment, elicit different characteristics of cell differentiation and factor release.

### 4.3. Limitations of the Chosen 3D Model System and the Comparison of 2D and 3D Results of Osteogenic Differentiation of MSC

The magnetic nanobead-based 3D spheroid model was chosen because the spheroid size can be precisely adjusted [21,22]. This way, the spheroids are created without a scaffold and initially have no extracellular matrix microenvironment. This initial lack means that the cell–extracellular matrix contacts and the range of elasticity of an extracellular environment are not initially offered. Both the composition of the extracellular environment and its physicochemical properties influence the behavior of MSC in terms of proliferation and differentiation [50,55]. The spectrum of suitable extracellular matrices for 3D cell culture is wide, and the various matrices differ in aspects such as molecular composition, elasticity, stiffness, etc. [56,57,58]. It would be interesting to combine the advantages of the different 3D models and, for example, incorporate the magnetic nanobead-equipped MSC into a scaffold that mimics the natural environment and then cultivate them in a spheroid.

A fundamental problem in interpreting the available data showed that the different culture systems (2D and 3D culture) exhibit large differences depending on the study design. Many aspects influence the variability of the results. For example, the respective MSC type (even subpopulations and cell donor-dependent differences can lead to differences [15,59]), the seeded cell numbers and thus the spheroid size, the composition of the cell culture medium and additives, the growth area or scaffold (if available), and the culture periods can have a significant influence on the final result [37]. In addition, long-term cultures of MSC tend to spontaneously form spheroids [60,61], which either continue to adhere to the culture dish bottom and coexist with the 2D culture or detach and are inadvertently removed with the following change of culture medium. This high level of variability can have a massive impact on the overall result. In addition, as described in Section 4.2, comparability is limited by the different detection methods used for osteogenic differentiation, as not all detection methods are generally applicable to all in vitro culture models. With the various 3D model systems, other components come into play that further complicate the situation. These can be magnetizable nanobeads, as used in our study, but also scaffolds or specially treated surfaces of natural, semi-synthetic, or synthetic material provided to initiate the 3D cell culture. All of these materials can potentially interact with the components under study, further impeding interpretability.

## 5. Conclusions

This study has demonstrated that the chosen magnetizable nanobead-based 3D spheroid model is a reliable tool for studying adMSC in a 3D environment, as it allows the generation of spheroids of a uniform cell number and size that can be studied over a comparatively long culture period of 35 days. The chosen 3D spheroid model ensures reliable osteogenic differentiation and bone-specific matrix deposition at a time point close to the physiological process of bone healing.

It must be emphasized that the explanatory power of such comparative studies is limited due to the different methods of analysis that have to be applied in 2D and 3D. Furthermore, an extensive technical effort must be made for a high-quality analysis of the 3D spheroids, which can only be implemented with a high level of expertise, so it is never a matter of quick results.

The results based on these experiments provide new insights into the effects of adMSC cell arrangement on osteogenic differentiation. Currently, there needs to be more information on the signal transduction and control of MSC survival and differentiation in 3D cultures. Therefore, further efforts are required to study the mechanisms and interactions in more detail, including understanding the processes when adMSC are introduced into a complex 3D environment during cell therapeutic applications. Further improvement in cell culture techniques in conjunction with new biotechnological approaches (e.g., organ-on-a-chip models) should also support the 3Rs principle to reduce animal testing.

## Figures and Tables

**Table 1:** Quantification of bone metabolism-affecting factors in cell culture supernatants of unstimulated (US) and osteogenically (OS) stimulated 2D and 3D spheroid cultures (multiplex assay, biological replicates: *n* = 4, technical replicates were involved in the calculations, median values in pg/10^4^ cells, descending order depending on detected quantity).

		2D		3D	
Analyte		US	OS	US	OS
**DKK1**	Day 7	164,301	2,507,609	214	4665
	Day 28	920,208	392,085	4319	4215
**OPG**	Day 7	7532	2415	25	6
	Day 28	4136	1046	35	10
**IL-6**	Day 7	6454	1976	663	24
	Day 28	2617	419	760	59
**SOST**	Day 7	1578	2148	0	0
	Day 28	574	472	0	0
**Leptin**	Day 7	13	691	0	0
	Day 28	36	1227	0	0
**OPN**	Day 7	57	67	0	0
	Day 28	28	17	0	0
**OC**	Day 7	42	62	0	0
	Day 28	18	19	0	0
**Insulin**	Day 7	0	19	0	0
	Day 28	0	7	0	0
**FGF23**	Day 7	0	17	0	0
	Day 28	1	7	0	0

## Data Availability

Data are contained within the article or Supplementary Materials.

## Supplementary Materials

The following supporting information can be downloaded at: https://www.mdpi.com/article/10.3390/biomedicines11041049/s1, Figure S1: Analysis of the spheroid diameter of unstimulated (US) and osteogenically stimulated (OS) spheroids; Figure S2: SEM-EDX analysis of unstimulated and osteogenically stimulated adMSC in 3D spheroid culture after 28 days; Figure S3: SEM-EDX spectrum analysis of unstimulated and osteogenically stimulated adMSC in 3D spheroid cultures after 14 and 28 days of cultivation; Table S1: Quantification of bone metabolism-affecting factors in cell culture supernatants and lysates from unstimulated (US) and osteogenic (OS) stimulated 2D and 3D cultures.
