# Supplementary material for: 3D Spheroid Cultivation Alters the Extent and Progression of Osteogenic Differentiation of Mesenchymal Stem/Stromal Cells Compared to 2D Cultivation
Source: Biomedicines. 2023 Mar 29;11(4):1049. doi: 10.3390/biomedicines11041049 (PMC10135665; doi:10.3390/biomedicines11041049)
Supplement: Supplementary file 1 [file biomedicines-11-01049-s001.zip › biomedicines-2254803-supplementary.pdf]

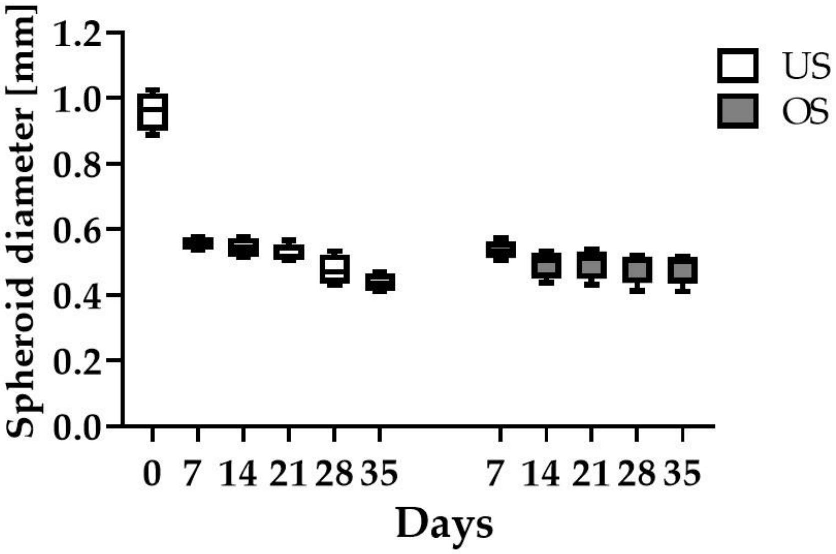

Figure S1. Analysis of the spheroid diameter of unstimulated (US) and osteogenically stimulated (OS) spheroids at day 0, and after 7, 14, 21, 28 and 35 days of cultivation (image analysis performed with ImageJ, n = 4).

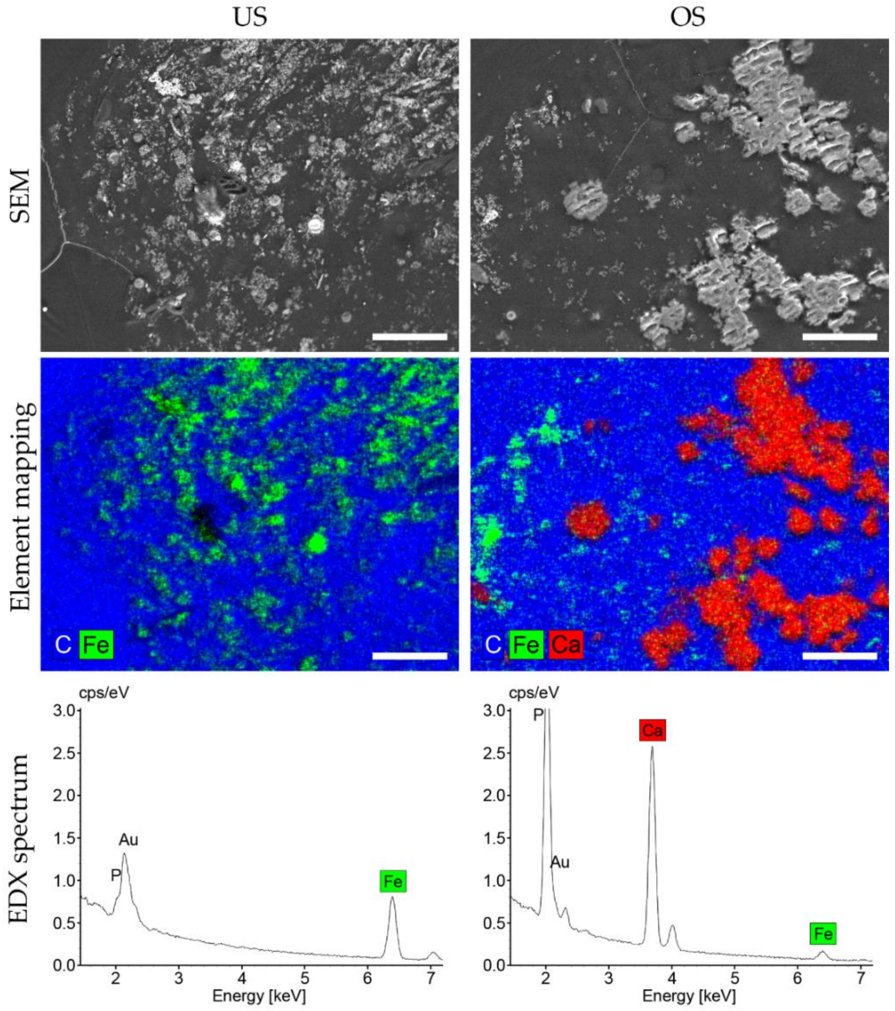

Figure S2. SEM-EDX analysis of unstimulated and osteogenically stimulated adMSC in 3D spheroid culture after 28 days. 'Block-face' imaging of the embedded unstimulated spheroids (US) or osteogenically stimulated spheroids (OS) was performed after histological sections were prepared. Imaging with secondary electron detection outlines structural features of the samples, i.e. metal particles and deposited calcified matrix, which are displayed as brighter areas (top panel). In combination with X-ray spectroscopy an elemental map is generated by assigning a color coding that shows iron (green) and calcium (red) embedded in a carbon containing matrix (blue), representing spheroid cells and resin (middle panel). The spectra obtained during the mapping (acquisition time 6 min) show that considerable amounts of calcium and phosphorus are exclusively found in the OS condition, indicating substantial matrix deposition of calcium-phosphate. Note that larger agglomerations of calcium containing matrix deposits tend to be formed by merging of individual spots compared to the condition at 14 days of culture (see Figure 6) (scale bars: 30 μm).

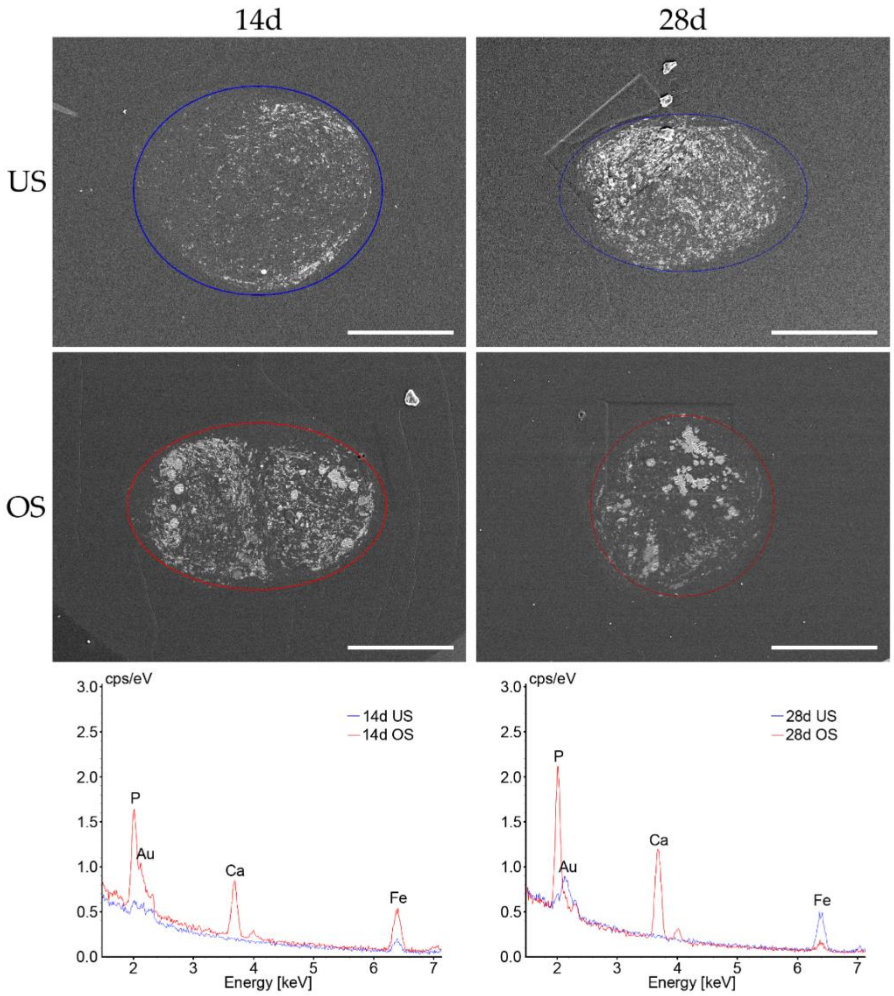

Figure S3. SEM-EDX spectrum analysis of unstimulated and osteogenically stimulated adMSC in 3D spheroid cultures after 14 and 28 days of cultivation. Spectrum recordings were obtained from the polished surfaces of the embedded spheroid blocks after cutting of the histological sections, a so-called 'block-face' condition. Characteristic element specific X-rays were detected across the total surface area of the respective unstimulated spheroids (US), encircled in blue color (top panel) or of osteogenic stimulated spheroids (OS) encircled in red color (middle panel) for a total of 3 x 10^5 counts using a 30 μm aperture and 12 kV acceleration voltage. The overlay of the resulting spectra clearly demonstrates that peaks identifying calcium and phosphorus are limited to the osteogenic conditions (red spectra) and are virtually absent from the unstimulated conditions (blue spectra) at both time points (bottom panel) (scale bars: 200 μm).

Table S1. Quantification of bone metabolism-affecting factors in cell culture supernatants and lysates from unstimulated (US) and osteogenic (OS) stimulated 2D and 3D cultures (multiplex assay, biological replicates: n = 4, technical replicates were involved in the calculations, represented as median and the respective minimum (Min) and maximum (Max) value, in pg/10^4 cells, in descending order depending on the amount detected, with decimal place in the single digit range).

Analyte			Supernatant						Lysate					
			2D			3D			2D			3D		
			Median	Min	Max	Median	Min	Max	Median	Min	Max	Median	Min	Max
DKK1	Day 7	US	164301	85757	307129	214	170	400	182	5,3	198	4,4	2	94
		OS	2507609	825760	2722471	4665	2636	5781	490	56	773	22	16	351
	Day 28	US	920208	851199	1112361	4319	69	35934	112	95	135	14	1,9	43
		OS	392085	284111	1032174	4215	1548	6250	299	145	417	25	14	38
OPG	Day 7	US	7532	6543	9860	25	13	28	120	1,6	239	17	1,3	94
		OS	2415	2317	3745	6,2	5,4	9,3	48	2,2	79	3,5	0,4	33
	Day 28	US	4136	3004	7668	35	0,1	84	230	132	1298	6,6	1,4	12
		OS	1046	653	8554	9,7	2,9	15	92	42	237	2,2	1,4	4,0
IL6	Day 7	US	6454	4504	9230	663	394	807	57	19	126	28	6,1	58
		OS	1976	1071	3053	24	20	40	4,0	0	9,2	2,4	1,3	15
	Day 28	US	2617	2403	3955	760	143	5102	51	8,8	199	0	0	0
		OS	419	224	2167	59	54	83	4,6	1,3	7,8	0,2	0	4,4
SOST	Day 7	US	1578	488	1879	0	0	0	0	0	0	0	0	0
		OS	2148	2058	2435	0	0	0	0	0	0	0	0	0
	Day 28	US	574	546	1135	0	0	0	90	72	236	0	0	0
		OS	472	394	1138	0	0	0	69	23	383	0	0	0
Leptin	Day 7	US	13	0	20	0	0	0	0	0	0	0	0	0
		OS	691	672	1158	0	0	0	0	0	0	0	0	0
	Day 28	US	36	8,4	170	0	0	0	0	0	0	0	0	0
		OS	1227	1029	3200	0	0	0	0	0	0	0	0	0
OPN	Day 7	US	57	53	146	0	0	54	0	0	0	0	0	0
		OS	67	57	83	0	0	0	0	0	0	0	0	0
	Day 28	US	28	23	69	0	0	183	0	0	0	0	0	0
		OS	17	7,6	49	0	0	65	0	0	0	0	0	0
OC	Day 7	US	42	0	119	0	0	26	0	0	0	0	0	0

		OS	62	48	171	0	0	0	0	0	0	0	0	0
	Day 28	US	18	9,4	55	0	0	0	0	0	0	0	0	0
		OS	19	8,5	63	0	0	0	0	0	0	0	0	0
**Insulin**	Day 7	US	0	0	0	0	0	0	0	0	33	0	0	0
		OS	19	3,0	28	0	0	0	0	0	0	0	0	0
	Day 28	US	0	0	0	0	0	0	2,4	0	14	0	0	0
		OS	6,9	0,5	10	0	0	0	0	0	11	0	0	28
**FGF23**	Day 7	US	0	0	0	0	0	0	0	0	0	0	0	0
		OS	17	3,7	25	0	0	0	0	0	0	0	0	0
	Day 28	US	0,7	0	5,8	0	0	0	0	0	0	0	0	0
		OS	6,5	5,5	10,0	0	0	0	0	0	0	0	0	0
**PTH**	Day 7	US	0	0	0	0	0	0	1,1	0,7	1,9	0,4	0,2	0,7
		OS	0	0	0	0	0	0	0,8	0,6	1,6	0,2	0,1	0,5
	Day 28	US	0	0	0	0	0	0	0,6	0,4	2,0	6,4	1,4	17
		OS	0	0	0	0	0	0	0,3	0,1	1,5	0,8	0,5	2,8
**IL1β**	Day 7	US	0	0	0,1	0	0	0	0,1	0,1	0,3	0	0	0
		OS	0,1	0	0,1	0	0	0	0,1	0	0,1	0	0	0
	Day 28	US	0	0	0	0	0	0	0	0	0,1	0,4	0	0,8
		OS	0	0	0	0	0	0	0	0	0,1	0,1	0	0,1
**ACTH**	Day 7	US	0	0	0	0	0	0	0	0	0	0	0	0
		OS	0	0	0	0	0	0	0	0	0	0	0	0
	Day 28	US	0	0	0	0	0	0	0	0	0	0	0	11
		OS	0	0	0	0	0	0	0	0	0.9	0	0	0,6
**TNFα**	Day 7	US	0	0	0	0	0	0	0	0	0	0	0	0
		OS	0,1	0,1	0,1	0	0	0	0	0	0	0	0	0
	Day 28	US	0,0	0	0	0	0	0	0	0	0	0	0	0
		OS	0	0	0,1	0	0	0	0	0	0	0	0	0
